# A review of required monitoring and management of physical health parameters in patients being treated with clozapine

**DOI:** 10.1192/bjo.2021.326

**Published:** 2021-06-18

**Authors:** Louisa Ward, Charlotte Marriott, Giles Glass, Mariam Negm, Hannah Porter

**Affiliations:** worcestershire health and care trust

## Abstract

**Aims:**

To review available standards for physical health monitoring in people taking clozapine To audit current practice against standards To identify changes in practice and facilitate a re-audit to assess impact of any changes

**Method:**

Standard: CG178 Psychosis and Schizophrenia in Adults: Prevention and Management – NICE, February 2014

Target:100%

Exceptions: None

Sample: The original audit included all 58 patients from the Worcester clozapine clinic, as per October 2018. The re-audit reviewed a random sample of all patients attending the clozapine clinics in Worcester, Kidderminster and Redditch, as part of Worcestershire Health and Care NHS Trust, as per October 2019. A total of 66 patients were selected.

Data Source: Carenotes and ICE

**Result:**

Areas of good practice:

Monitoring of HbA1c and FBC remains good

There has been an improvement in monitoring alcohol use, substance misuse and side effects

Areas requiring improvement:

There continues to be limited recording of respiratory rate

There has been a decline in recording temperature, BMI and concomitant therapies

Potential reasoning for missing data includes:

Staff not knowing the monitoring requirements, which is more likely to be an issue when staff members running the clinics change frequently

Monitoring being completed but not documented

Patients’ refusal of monitoring

Data being recorded in alternative locations including general practice, without communication between services

Patients moving between teams or having inpatient stays may disrupt monitoring regime

**Conclusion:**

LIMITATIONS

This audit assumes all patients involved to be on a stable dose of clozapine with routine monitoring

Some patients may have been transferred between teams or inpatients during the period of data collection

There is no scope to record when patients refuse monitoring

We may not have access to all notes such as those from general practice for data collection

RECOMMENDATIONS

Induction programme for junior doctors to include education on clozapine monitoring

Training for staff involved in clozapine clinics to ensure better understanding of monitoring requirements

Procurement of ECG machines for each site and relevant training for nursing and medical staff

Collaboration with GPs for shared data

Re-audit in 1 year

